# Design Meets Neuroscience: An Electroencephalogram Study of Design Thinking in Concept Generation Phase

**DOI:** 10.3389/fpsyg.2022.832194

**Published:** 2022-03-03

**Authors:** Ying Hu, Jieqian Ouyang, Huazhen Wang, Juan Zhang, An Liu, Xiaolei Min, Xing Du

**Affiliations:** ^1^School of Design, Hunan University, Changsha, China; ^2^School of Statistics, Capital University of Economics and Business, Beijing, China; ^3^College of Furniture and Design, Central South University of Forestry and Technology, Changsha, China

**Keywords:** design cognition, EEG, design research, design process, research methods

## Abstract

Extant research on design thinking is subjective and limited. This manuscript combines protocol analysis and electroencephalogram (EEG) to read design thoughts in the core design activities of concept generation phase. The results suggest that alpha band power had event related synchronization (ERS) in the scenario task and divergent thinking occupies a dominant position. However, it had event related desynchronization (ERD) in analogy and inference activities, etc., and it is stronger for mental pressure and exercised cognitive processing. In addition, the parietooccipital area differs significantly from other brain areas in most design activities. This study explores the relationship of different design thinking and EEG data, which is innovative and professional in the field of design, providing a more objective data basis and evaluation method for future applied research and diverse educational practices.

## Introduction

Design thinking is generally defined as an analytic and creative process (Kaye, 2006) that is considered a key means to generating novel ideas and innovation (Martin, 2009; Deserti and Rizzo, 2014). In order to develop effective design method and improve current design technology (Zhu et al., 2007; Nguyen and Zeng, 2012), it is necessary to analyze the design thinking of design activities.

The most frequently used technique in design research is verbal protocol analysis (Nguyen and Zeng, 2012), but this technique is quite subjective as it only enables us to observe the observable or describable behaviors (Nguyen and Zeng, 2012), and the results are hard to verify. Therefore, a new technique is needed to help researchers achieve more objective and verifiable reading of design thoughts.

With the development of cognitive neuroscience, EEG provides the technical means to exploring the brain stimulus modes (Arden et al., 2010; Jung and Oshin Vartanian, 2018) and supporting other researches (Bowden and Jung-Beeman, 2007; Fink et al., 2007; Luo and Knoblich, 2007; Srinivasan, 2007). With EEG, researchers can observe the changing physiological signals to study the change of thinking in the brain (Göker, 1997; Alexiou et al., 2009), making the reading of design thoughts more objective and verifiable.

### Application of Electroencephalogram in Thinking-Related Research

Electroencephalogram technology, a method of recording brain activities that detects electrical signals that reflect the signal flow in the brain, is important for revealing the neurological basis for individual cognitive and thinking activities (Shen et al., 2010). The neurological basis for creative thinking is an important area of creativity studies (Schwab et al., 2014), and a great deal of scientific research has been done, with the help of EEG technology which is highly time-sensitive (Dong et al., 2004), in the attempt to find out the brain stimulus mode of creative thinking (Arden et al., 2010; Jung and Oshin Vartanian, 2018). As the key to creative thinking, design thinking is of great research value.

A lot of studies in recent years have adopted neuroscientific methods, some of which focus on originality, novelty, insight, divergent thinking, and other processes related to creative mental activity (Runco and Yoruk, 2014). EEG studies have revealed that signals in several frequency bands, such as the θ (4–8 Hz), α (8–13 Hz), and β (13–30 Hz) bands, are associated with creative thinking (Dietrich and Kanso, 2010) (see Table 1). Some researchers have reported the frontal α increases in synchrony with divergent thinking (Fink et al., 2006, 2009a,b; Grabner et al., 2007), and the α at the temporal or parietal sites decreases as the originality score increases (Razumnikova et al., 2009).

**TABLE 1:** Comprehensive analysis of elements of thinking and EEG (frequency band, brain area, and traits).

Thinking EEG	Frequency bands				Area					Features	Methods

		α	θ	β	γ	Anteriofrontal	Parietotemporal	Centrotemporal	Temples	Occipital	Spectral power	Task-related power (TRP) (ERD/ERS)
Figural creative ideation	√					√			√	√	√

Divergent thinking	√				√	√		√		√	√

Convergent thinking	√	√			√					√	√

Insight			√	√	√		√			√	√

Reflection	√				√					√	√

Mindfulness meditation				√	√					√	√
											

Creativity during problem solving depends on random generation and validity test (Sweller, 2009; Sweller et al., 2019). Most of the electrophysiological knowledge about creative ideation/divergent thinking is based on verbal creative ideation tasks, e.g., the alternate uses task (AUT) (Arden et al., 2010; Fink and Benedek, 2014). On the basis of such tasks, this manuscript assigns design tasks as the participants’ thinking tasks and studies and records the designers’ state when conducting the tasks. We coded the participants’ oral expressions of their thinking process to further analyze their thinking state during design activity.

Changes of EEG activity in different frequency bands have been observed to reflect various aspects of cognitive activity (Christa and Wolfgang Klimesch, 2006). The power increase in event-related frequency bands is called event related synchronization (ERS) (Benedek et al., 2011), while power decrease is called event related desynchronization (ERD) of the EEG. The ERD/S method has been widely used in different research fields of cognitive needs (Klimesch, 1999; Klimesch et al., 2007). Besides, the reliability and internal consistency of ERD was about 0.8 (Burgess and Gruzelier, 1996; Fink et al., 2005), which indicates “excellent” reliability according to common biostatistical classifications (Cicchetti and Sparrow, 1981). This manuscript records and observes the designers’ EEG data in different designing tasks in comparison to the reference state, analyzes the power synchronization and desynchronization in different frequency bands and identifies the EEG presentation of thinking process during different design activities.

### Design Activity in Concept Generation Phase

In the designing process, some of the most advanced human cognitive abilities are employed, such as creativity and problem-solving ability (Cross et al., 1996). Articles and services are used as medium to establish the design itself and the system it is in to enhance the overall design quality. Design activity refers to a series of meaningful operations by the designer to conceptualize his/her design thoughts (Chen and Venkatesh, 2013) and push the design from the current state to a new state (Jin and Ishino, 2006). The activity theory has been extensively employed in the field of psychology (Falikman and Asmolov, 2017), education (Bligh and Flood, 2017; Hancock and Miller, 2018), and man-machine interaction (Clemmensen et al., 2016) to understand the systematic activities of individuals or groups. In design, activity theory is applied in two ways: as a design tool or a qualitative framework (Zahedi et al., 2017). As a design tool, activity theory is used in various subjects to help identify problems and conflicts associated with specific projects or scenarios. As a qualitative framework, it is used for evaluation either during or after the project to study the designing process and choices of the design team or various variables that may affect the design results. Many existing studies have discussed design activity to analyze design thinking (Samuel and Sobek, 2004; Jin and Ishino, 2006; Pedgley, 2007; Luck, 2010; Cash and Kreye, 2018). It’s clear that design activity studies can help understand the invisible activity of design thinking. This manuscript uses design activity as a medium to collect the EEG traits of the participants based on their performance during specific design activities.

The design process as a phenomenon has been a topic of design research (Sim and Duffy, 2003). It is important to quantify the designer’s cognitive processes to develop an effective, structured and logical design methodology (Nguyen and Zeng, 2010). In the whole designing process, the concept generation phase is characterized by the most active and creative design thinking (Hu et al., 2019), during which the designer’s design activity can be generalized into four stages: naming, framing, moves, and evaluating (Dorst, 1997). We developed 18 design activity codes more suitable for the concept generation phase according to the general coding system for design thinking developed by Cross and Dorst (Hu et al., 2019). Considering that a long-lasting experiment would fatigue the participants and consequently affect the EEG signals, we selected 7 core design activities from the 18 codes according to their importance and frequency based on our previous research (Hu, 2014), and observed the participants’ EGG data. With this and the protocol analysis, we obtained the EEG traits of design thinking for different design activities, thus contributing to studies of designing process and methods. Few other studies have used design activity codes to analyze the design process (Fink et al., 2006, 2009a,b; Grabner et al., 2007). With references to relevant literature, this manuscript is a more detailed, innovative and professional.

## Materials and Methods

### Participants

Twelve individuals (6 male and 6 female) recruited online participated in the experiment. All of them were junior or higher-level students majoring in design. The age of the participants ranged from 21 to 25 (*M* = 22.84; *SD* = 4.64) and they were all right-handed as the task was based on the research on righties (Steingrüber and Lienert, 1971; Ilona and Schulter, 1999); non-medicated, informed and written consent was obtained. Participants were requested to refrain from alcohol, coffee and other stimulating beverages for 24 h prior to their lab appointment, and to come to the session well rested. They were also made aware of a certain amount of compensation after the experiment. The experiment conformed to the Declaration of Helsinki. The study was approved by the ethics committee of Central South University of Forestry and Technology and School of Design, Hunan University.

Considering that a long-lasting experiment would fatigue and bore the participants and consequently affect the reliability of EEG data, each experiment was kept as short as possible. Before the experiment officially began, our staff conducted a pre-testing. Each experiment was kept within 3 min in order to strike a balance between the results and the participants’ state, and the participants were given enough time to rest and refresh during the intervals.

### Experimental Task

To accurately mark design activity in different phases, identify the EEG traits of design thinking for different design activities and further observe the changes in design thinking, we explained the 7 core design activities (Hu et al., 2019)that have been extracted (see Table 2). We marked the EEG data for each designing task and collected data both for the task state and the reference state.

**TABLE 2:** Design activities and definitions.

Design activity	Definition
Scenario establishment	Establishing the scenario of design problems
Scenario shift	Shifting among multiple scenarios
Problem defining	Defining design problems, determining limitations, principles, and rereading design requirements
Analogy and inference	Creating a new design plan in reference to existing cases (user needs, design plan, commercial mode, etc.)
Synthesis	Synthesizing multiple existing concepts (created by the designer or others) into a new plan
Mutation	Creating a new plan free from all references
Reflection	Does the plan meet the limitations and design principles? Is it meaningful?

Scenario establishment and scenario shift were accomplished through three different designing tasks: imagining a forest-themed natural scenario, imagining contents for the title “receiving short message,” and imagining a specific scenario titled “studio.” We tried to find EEG traits of scenario establishment and scenario shift.

**FIGURE 1:**
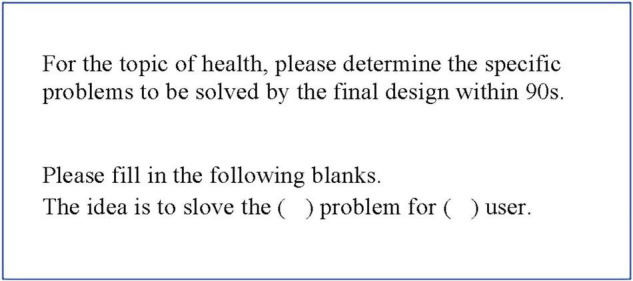
Task information for problem defining.

For the task of problem defining, after determining health-themed (see Figure 1) design problems, the participant was asked to write down value assertions (see Figure 1) to unify the problems to be defined, and generated an idea according to the confirmed value assertions.

For the task of analogy and inference, the participant was asked to create a new idea in reference to existing cases. After the participant generated an idea, we would provide them with a similar case in the stage of reading task information on analogy and inference. After reading the task information, the participant had to infer a new idea based on the case provided.

For the task of synthesis, the participant was asked to add a new element provided by the experimenter to his/her plan. The experimenter, based on the participant’s current plan, would randomly choose one of the following new elements: socializing, emergency contact person or accompanying, and ask the participant to integrate it in their idea.

**FIGURE 2:**
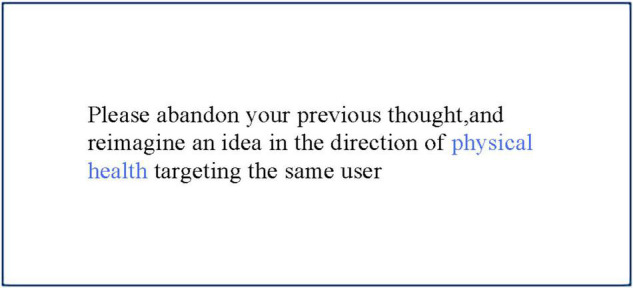
Task information for mutation.

For the task of mutation, the participant was asked to abandon all the reference contents and ideas and come up with a completely new idea (see Figure 2), targeting the same user but in another theme.

For the task of reflection, the plan’s feasibility was considered. The participant evaluated the feasibility of their current idea according to the value assertions.

### Data Acquisition

**FIGURE 3:**
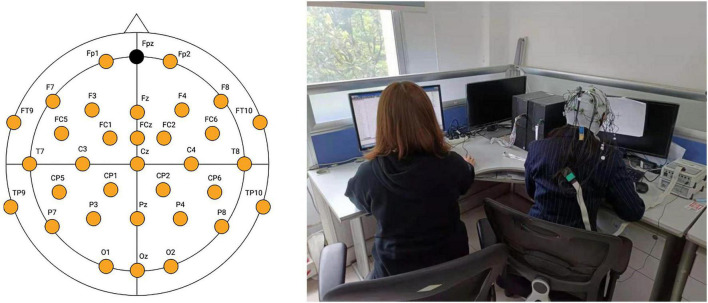
EEG electrode positions (left) and experiment site (right).

We set up the experiment according to some existing cognitive experiments (Nguyen and Zeng, 2012; Cruz-Garza et al., 2020; Salvi et al., 2020; Stevens and Zabelina, 2020). After the participant put on an electrode cap (BrainProduct actiChamp-32) in 33 positions (according to the international 10–20 system with interspaced positions) (Liu et al., 2018), the product’s electrode impedance was tuned down to below 10 kΩ with Brain Vision Recorder. Then EEG data began to be recorded at the equipment frequency of 1000 Hz, with the electrode positions and experiment scenarios shown in the left and right part of Figure 3.

Before the experiment, we turned off all other electronic equipment than the experiment equipment to ensure that the room remained quiet and bright to create a suitable experimental environment. The participant (wearing the electrode hat, right of Figure 3) was sitting in a comfortable chair in a quiet and sound-proof room lit with soft light, while the experimenter (observing and recording EEG data, right of Figure 3) was sitting in front of a computer on the left back of the participant, where it was easy to observe the real-time EEG data. When the experiment began, the participant was asked to find a thinking-friendly sitting posture as comfortable as possible and not to move during the experiment.

### Experimental Procedure

**FIGURE 4:**
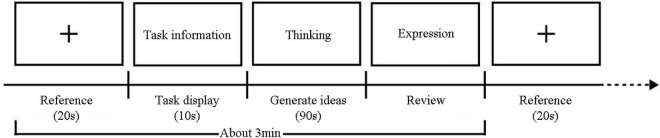
Experiment duration and procedures.

The participant should operate and think according to the questions on the tablet, and might gesture to the experimenter every time they finished reading a question if they had questions or was ready to begin thinking. Every section was followed by a relaxing period. When there was no big fluctuation in EEG for 10s, the participant was in the right state to move on to the next part. Each designing task lasted about 3 min and the whole experiment lasted less than 90 min, including time for equipment commissioning. The experimental procedures were based on the regular process of EEG experiment (see Figure 4).

Reference: 20s relaxation; Task information: 10s display of task information; Thinking: 90s idea generation; Expression: retrospective description of the thinking process in concept generation phase.

Every participant had to complete ten different designing tasks, including three different scenario tasks and one task of conceptual design. Tasks such as reflection, mutation and analogy and inference had to be conducted after a plan was established.

The EEG-recorded experiment lasted 120 s. After the equipment was worn and commissioned, the participant would enter a 20-s relaxing phase as per the equipment’s reminding and was asked to generate ideas in mind for 90 s, like in an AUT task (Fink et al., 2011, 2012). After completing the thinking process, the participant would orally communicate the process to the experimenter in the expression phase, and the experimenter would record the oral description with sound recording and video.

### Retrospective Coding

Considering the complexity and uncontrollability of design thinking, we collected the audio of each design expression, and used protocol analysis as the main method of voice coding to more accurately identify design thoughts during design tasks.

Data was coded based on the following pre-defined design activities: search for problem definition, analogy and inference, synthesis, mutation, reflection and search for solution, scenario establishment and shift.

Two researchers with more than 10 years’ design research experience and more than 6 years’ coding experience coded the data. We performed Kappa comparison of the coding results, and finally achieved a high degree of fit (0.81), which means the coding results were reliable. Any discrepancies in the coded data were discussed and resolved (Nguyen and Zeng, 2012).

We collected 120 pieces of audio data, each lasting about 90 s. They were coded by the researchers according to the experimental design tasks to determine the data validity and valid duration (see Table 3). Retrospective coding showed that the participant’s thinking didn’t follow the designing task requirement all the time, but was complex and constantly changing. The participant (see Table 3) did not reflect all the way through the reflecting stage, but went through the following thinking stages: reflection, conceptual design, reflection, and conceptual design. We inferred the time frames of the participant’s reflecting activity when performing the design task according to the duration of their oral description. Moreover, retrospective coding also helped us know more about the participant’s motive and thinking process so as to better understand the EEG changes.

**TABLE 3:** Results of retrospective voice coding in reflection stage.

Reflection				
Start	End	Video Data	Code	Video data
**0:35:56**	0:36:04	Causing extra cost to the company is absolutely out of the question	Reflection	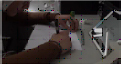
**0:36:04**	0:36:15	Other aspects are quite feasible. Then I thought about how to control this extra expense. The reward can be offered once a week – but this is a preliminary idea	No code	
**0:36:15**	0:36:24	Another problem with this idea for employees is that the capsules are random. The capsules they take may not be the right ones	Reflection	
**0:36:24**	0:36:53	So I still want to provide a way to change capsules. Then I thought that since it’s a company, there must be performance ranking. You can change the frequency to once a week, or once a month. What’s the benefit for those ranking atop? For example, if you are the first, you can choose capsules directly. If you are the second, you will have a chance to change capsules. If you are the third, just forget about it. It’s based on their performance	No code	

### Data Collection and Pre-processing

After the experiment, we preliminarily screened the data results, excluding those with strong noises. Then we pre-processed the collected EEG data of 12 participants. We first used Brain Vision Analyzer 2.1 to analyze and screen the interference in original data, and then pre-processed the data. Bilateral mastoid electrodes (Tp9 and Tp10) were used for reference, independent component analysis (ICA) was used to remove eye-blink artifacts with the reference to Fp1 or Fp2 electrodes in the anteriofrontal area (Jung et al., 2000), and infinite impulse response (IIR) Filters (low pass filter) was adopted for 0–45 Hz filtering. Baseline data and task-state data were separated through manual marking during the experiment, and they were matched with the retrospective interview coding of each task to obtain the baseline data of each participant as well as the corresponding task-state data.

After the EEG data of the 12 participants were pre-processed, we screened them in view of the coding of retrospective interviews, and obtained the following valid data: 10 data sets for synthesis, 16 sets for reflection, 9 for naming, 16 for scenario tasks and 10 for inference. Each data set includes 20-s resting-state data and 90-s task-state data. It is worth noting that the experimental process and tasks of all participants are the same. But in reality, design activities beyond task setting often appeared when they were thinking. This is why we use coding of retrospective interviews to improve reliability. Due to the randomness of the appearance, there will be a little difference in the amount of different design activity codes.

### Event Related Desynchronization/Event Related Synchronization Analysis

We extracted five frequency bands from the pre-processed EEG signals by fast Fourier transform (FFT) with Hanning window, namely δ (0.5–3.5 Hz), θ (3.5–8 Hz), α (8–13 Hz), β (13–30 Hz), and γ (31–45 Hz), and calculated the power in corresponding state with Fourier transform. The changing TRP in EEG can quantify brain activities during the experiment (Pfurtscheller and Lopes da Silva, 1999; Liu et al., 2018). TRP is the result of the logarithm of task-state power minus the logarithm of reference power before the design task.

TRP (log Powi) = log [Powi activation] − log [Powi reference].

In TRP analysis, we calculated the power change of every electrode in task state in comparison to the reference period, which was the marked 20-s period. Negative values indicate a decrease of task-related power from the reference to the activation period, while positive values express a power increase (Pfurtscheller and Lopes da Silva, 1999).

## Results

Considering the complexity and diversity of design thinking, we analyzed every independent and explainable design task rather than the entire designing process. EEG data were collected for each design activity and analyzed in reference to the retrospective voice coding to identify design thinking more accurately and objectively. The results are as follows.

### Coding Results

Retrospective coding showed that participants were in a relaxed state during scenario task, but they were rarely in a single mutation state during the mutation task, when the most frequent combination was “problem defining + mutation” or “reflection and search for solution + mutation.” The coding result indicated that designers tended to enter the mutation state from the stage of problem defining or reflection and search for solution, but they couldn’t sustain the mutation state for long.

### Electroencephalogram Results

**FIGURE 5:**
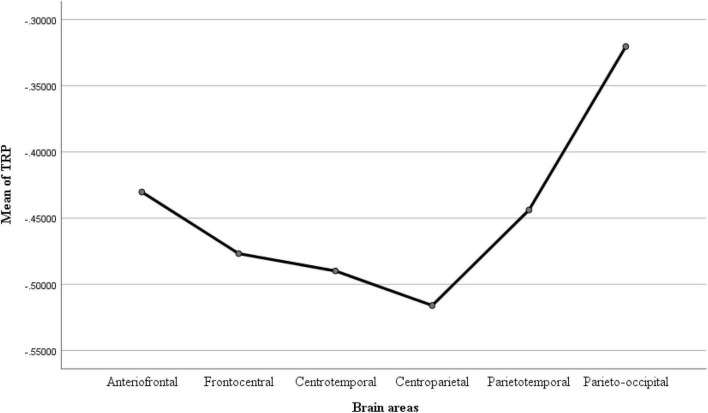
Mean of TRP in different brain areas.

After the tests of normality, we find that except for the problem defining, other codes do not conform to the homogeneity of variance. In order to solve this problem, the analysis method of assuming non-uniformity of variance in Statistical Package for Social Science (SPSS) is used in the next one-way ANOVA. The results of analysis of variance (ANOVA) analysis for different codes and different brain areas (average value of the electrodes in corresponding brain areas, see Figure 5) are shown in Tables 4, 5. From Table 4 we can see that there is significance in TRP values between different design activities (*p* = 0.000 < 0.05), so it is feasible to conduct ANOVA analysis on a single code. Table 5 shows significance in TRP values between different design activities in different brain areas (*p* = 0.000 < 0.05), so ANOVA analysis can be carried out, respectively, in different frequency bands.

**TABLE 4:** TRP ANOVA in different design activities.

ANOVA					
Dependent variable: TRP					
	Sum of squares	df	Mean square	*F*	Sig.
Between group	190.703	4	47.676	63.248	0.000
Within group	1353.063	1795	0.754		
Total	1543.766	1799			

**TABLE 5:** TRP ANOVA in different brain areas.

ANOVA						
Dependent variable: TRP		Sum of squares	df	Mean square	*F*	Sig.
Anteriofrontal	Between groups	36.782	4	9.196	11.122	0.000
	Within groups	243.908	295	0.827		
	Total	280.691	299			
Frontocentral	Between groups	36.756	4	9.189	11.134	0.000
	Within groups	243.463	295	0.825		
	Total	280.219	299			
Centrotemporal	Between groups	37.097	4	9.274	12.329	0.000
	Within groups	221.909	295	0.752		
	Total	259.006	299			
Centroparietal	Between groups	37.788	4	9.447	12.184	0.000
	Within groups	228.724	295	0.775		
	Total	266.512	299			
Parietotemporal	Between groups	31.033	4	7.758	10.960	0.000
	Within groups	208.819	295	0.708		
	Total	239.851	299			
Parieto-occipital	Between groups	17.138	4	4.284	6.542	0.000
	Within groups	193.209	295	0.655		
	Total	210.347	299			

**FIGURE 6:**
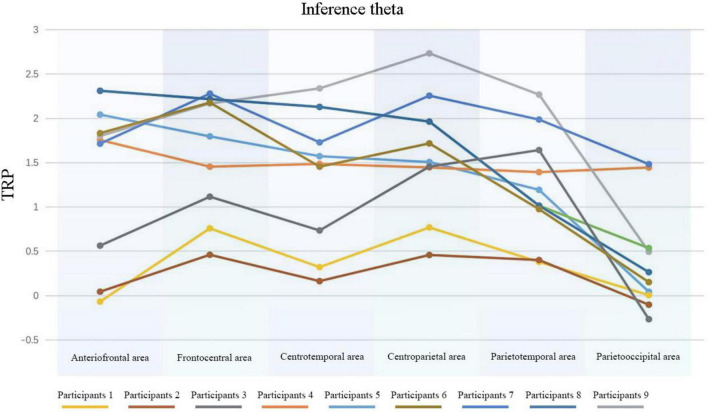
Analogy and inference: theta-band TRP. Each line represents the data of 9 different participants in different area.

Then we also conducted ANOVA on brain area in different bands (see Table 6) and analyzed the relation between different brain areas with Bonferroni multiple comparison. During the task of analogy and inference (see Table 7), the parieto-occipital area was found to have the most notable changes compared to other areas (*p* = 0 < 0.01). When we combined the ANOVA results with the calculated TRP data for each task (see Figure 6), the smallest TRP value appeared in parietooccipital area and mostly positive, while theta band displayed ERS in the task of analogy and inference.

**TABLE 6:** Analogy and inference: theta-band ANOVA.

Tests of between-subjects effects					
Dependent variable: TRP					
Source	Type III sum of Squares	df	Mean square	*F*	Sig.
Corrected model	63.100^a^	15	4.207	21.874	0.000
Intercept	200.980	1	200.980	1045.093	0.000
Hemispheres	1.621	1	1.621	8.428	0.005
Areas of brain	21.470	5	4.294	22.329	0.000
Group	40.009	9	4.445	23.116	0.000
Error	20.000	104	.192		
Total	284.080	120			
Corrected total	83.100	119			

*Brain areas: F = 22.329, p = 0 < 0.01, η2 = 0.518. There is a high level of significance, which means brain areas have significant impacts on theta activity (which differs significantly from one brain area to another) in inference task.*

*Hemiencephalon: F = 8.428, p = 0.005 < 0.01, η2 = 0.075. There is significance, which means hemiencephalon has significant impacts on theta activity in inference task.*

**TABLE 7:** Analogy and inference: theta-band Bonferroni multiple comparison.

Bonferroni						
Dependent variable: TRP						
(I) Areas of brain	(J) Areas of brain	Mean difference (I-J)	Std. error	Sig.	95% confidence interval	
					Lower bound	Upper bound
Parieto-occipital	Anteriofrontal	−1.02542304*	0.138675371	0.000	−1.30042151	−0.75042458
	Frontocentral	−1.25912857*	0.138675371	0.000	−1.53412703	−0.98413010
	Centrotemporal	−1.00036458*	0.138675371	0.000	−1.27536305	−0.72536612
	Centroparietal	−1.22157663*	0.138675371	0.000	−1.49657510	−0.94657817
	Parietotemporal	−0.82141140*	0.138675371	0.000	−1.09640986	−0.54641293

Based on observed means. The error term is Mean Square (Error) = 0.192. *The mean difference is significant at the 0.05 level.

Frequency domain analysis (Wang and Zhou, 2000) is a main analytical method of EEG application, with power spectrum estimation (Feng, 1986) being the chief means. With the help of Brain Vision Analyzer 2.1, this manuscript calculates the power spectral density (PSD) for five frequency bands [δ (0.5–3.5 Hz), θ (3.5–8 Hz), α (8–13 Hz), β (13–30 Hz), and γ (31–45 Hz)], and also calculates the task-related power (TRP) change on electrodes through Fourier transform (Pfurtscheller and Lopes da Silva, 1999; Liu et al., 2018). We also used SPSS to conduct ANOVA on left and right hemisphere and different areas (anteriofrontal, frontocentral, centrotemporal, centroparietal, parietotemporal, and parietooccipital) (Fink and Benedek, 2014). The results of frequency band and brain area analysis during different designing tasks are provided below (the data of scenario shift task were damaged and consequently not analyzed).

Although analysis of experimental data on scenario task showed that brain area didn’t have a significant impact on alpha activity [*F*(5,170) = 0.081, *p* = 0.995 > 0.05, η2 = 0.002], most participants’ alpha band power had ERS, implying a power increase compared to the resting state.

Analysis of experimental data on problem defining, analogy and inference, synthesis, and reflection and search for solution showed ERD of alpha band power, indicating a power decrease compared to the resting state.

During synthesis task, hemisphere had significant effect on beta band activity [*F*(1,104) = 21.169, *p* = 0 < 0.01, η2 = 0.169), so did brain areas (*F* = 311.893, *p* = 0 < 0.01, η2 = 0.364). Data showed that the minimal value of most TRP data in right central-temporal area was negative. The results showed a drastic decrease in beta band power in right central-temporal area during synthesis task.

During reflection task, brain area had significant impact on delta band [*F*(5,170) = 3.946, *p* = 0.002 < 0.01, η2 = 0.104) and gamma band [*F*(5,170) = 7.226, *p* = 0 < 0.01, η2 = 0.175), and the fluctuation in both bands displayed ERD. The results showed power decline in delta and gamma bands during reflection task.

During analogy and inference task, brain area had significant impacts on theta band [*F*(5,104) = 22.329, *p* = 0 < 0.01, η2 = 0.518), and Bonferroni multiple comparison indicated that the parietooccipital area displayed the most obvious changes (most significant difference) compared with other areas. Theta band fluctuation displayed ERS.

Additionally, we conduct t-test on the TRP of alpha band during synthesis, naming and inference tasks and found that most of data displayed ERD (*M* = −0.566 ± 0.059, *p* = 0.000 < 0.05).

## Discussion and Future Work

### Cognitive Processing of Designing Creation

Some studies showed that alpha band power would increase during creative divergent thinking (Fink et al., 2011) and we found a power increase in alpha band during scenario task. Besides, in view of the analysis of participants’ retrospective voice coding, we found that during a scenario task, participants would establish the scenario with remote associations based on the given elements, which was consistent with creative divergent thinking.

Alpha band power decrease is related with such cognitive processing activities as mental pressure, psychological effort, task difficulty, and degree of concentration (Ray and Cole, 1985; Klimesch et al., 2000; Fink et al., 2005). The more significant the power decrease, the higher the task requirements and the greater the participants’ psychological efforts (Gevins and Smith, 2005). With the power decrease in alpha band during problem defining task, we found that for this type of task, the participants would perform creative thinking based on their own cognitive resources in view of the analysis of participants’ retrospective voice coding, which, compared with divergent tasks, required more psychological efforts and cognitive processing.

Mólle et al. (1996, 1999) discovered that during convergent thinking, alpha power would decrease while theta power would increase in the frontal area. Participants’ retrospective voice data showed that the analogy and inference task during establishment-oriented artistic creation would trigger convergent thinking. The participants had to integrate the information about the provided design cases, refer to design points in those cases during inference, and conduct directed thinking and creation in sustained concentration.

### Summary

To find out the changes of design thinking, we extracted 7 core design activities from the participants’ designing process, studied their EEG traits, and read design thinking of each activity, thus laying the foundation for further study of thinking changes. By analyzing the experimental data and EEG data on 12 designers, we found, in addition to the EEG traits of corresponding design activities, that each design activity corresponded to changes in one or more brain areas and waveforms, and that some task traits had something in common in the midbrain area, with alpha and gamma waves changing most notably in the parieto-occipital area. Relevant studies indicated that visual α oscillations represent the gross disinhibition of visual processing circuits (Klimesch, 2012; Wiesman and Wilson, 2019; Wiesman et al., 2019), whereas visual γ activity has been associated with more fine-tuned stimulus encoding (Bertrand and Tallon-Baudry, 2000; Vidal et al., 2006; Edden et al., 2009). Furthermore, the posterior parietal cortex has been shown to be involved in various cognitive tasks (Simon et al., 2002; Walsh, 2003).

The research data showed that alpha band had significant differences between the parietal area and other brain areas during the task of synthesis, naming and inference, and most of the tested data showed ERD in alpha fluctuation. Neuroimaging research proved that visual memories contained in occipital cortex including primary visual area could be decoded, and graphic thinking research displayed a connection between the high visual processing requirements on the psychological generation of primitive and creative painting and the drastic decrease in parietooccipital alpha power (Pidgeon et al., 2016; Rominger et al., 2018). However, verbal creative thinking (e.g., during the AUT) was found to be associated mainly with increased α power at frontal and posterior cortical sites, reflecting top-down sensory control, bottom-up stimulation, and suppression of dispersive information flow from the visual system (von Stein and Sarnthein, 2000; Jensen et al., 2002; Crespo-Garcia et al., 2013; Fink and Benedek, 2014). Yet our experimental results indicated ERD in parietooccipital alpha power instead of drastic decrease. This might be because the designers, when performing the synthesis, naming and inference tasks, were good at mobilizing their visual work memories, during which AUT inhibited visual processing, hence the weak ERD of parietooccipital alpha power.

Gamma oscillations in neural circuits have long been hypothesized as a mechanism to facilitate the transient integration of distributed neuronal ensembles enabling cognitive functions such as attention and perception in different sensory modalities (Singer, 1999; Fries, 2009; Tallon-Baudry, 2009). And the gamma increases in more posterior visual areas are known to be modulated by a number of fine-tuned stimulus features and also by attention (Womelsdorf et al., 2006). Our studies showed that gamma band power had ERD in parietooccipital area during reflection and inference tasks, but had ERS in that area during synthesis task, from which we inferred that similar stimulus happened during reflection and inference, which was what happened with the participants.

Our research results are of positive significance to design education and refinement of design thinking activities.

1. Due to the diversity and complexity of design thinking, it is very challenging to analyze the thinking state of designers in design activities. Through the EEG features we found that this study obtained the differences between different design thinking codes, and explored the relevant neural basis. In design education, the research results point at a more objective and scientific design evaluation method compared with the previous ones. The feasibility of individual difference research based on our results makes it possible for educators to teach students according to their special design thinking mode, thus improving teaching efficiency.

2. This study induced specific design thinking activities through experimental settings and found EEG features, which can be used for further research on real design activities. In the process of design, people often use a variety of thinking modes at the same time, which is one of the reasons for the complexity of design thinking. The research results in this manuscript can gradually simplify the research on design thinking with detailed extraction and analysis of specific thinking code. It opens up a new way to the research on design thinking.

Taken together, our study indicated that during design activities, the designers’ design thinking mostly took place in the parietooccipital area, which was an important area for visual function and cognitive control. However, as the equipment had to be attached to the scalp to collect EEG signals, which was different from the normal designing or creative environment, the participants might feel nervous, stressed or otherwise psychologically distracted, which might affect the experimental results.

Moreover, the participants were students with limited design experience who could be described as novice designers. Studies of parallel cognitive behaviors divided novice’s cognitive behaviors into multiple parallel sets (Kavakli and Gero, 2002), but in our experiment, one participant’s EEG data displayed abnormally drastic volatility, and this set of data had to be abandoned despite our efforts otherwise. However, our interview in the later stage indicated that the said participant’s thoughts indeed changed very rapidly, which consisted with previous studies and also offered a possible direction for our future study of novice and expert designers. Meanwhile, although the sample size in this manuscript is not particularly large due to the reference of some existing experimental settings, the results are convincing enough. Expanding the sample size may help the exploration of our future research.

Going forward, we will refine the experiment to make the design activities more accurate, so as to better pinpoint the commonness and difference of the EEG traits of design thinking in different design activities. More experiments are needed to verify whether stimulation and modulation of the parietooccipital area through design activity can be conducive to the treatment of brain damages and how to evaluate the effects of simulation. Meanwhile, the law of occurrence and significance of different design activities is also one of the key points of the future research. Our research lays the foundation for identifying design thinking, opens up a new research path and expands the application of EEG in the field of design. It provides the potential direction and basis for the study of non-invasive brain-related visual processing and the measures for cognitive recovery and treatment.

## Data Availability Statement

The original contributions presented in the study are included in the article/supplementary material, further inquiries can be directed to the corresponding author.

## Ethics Statement

The studies involving human participants were reviewed and approved by the Ethics Committee of Central South University of Forestry and Technology and School of Design, Hunan University. The patients/participants provided their written informed consent to participate in this study. Written informed consent was obtained from the individual(s) for the publication of any potentially identifiable images or data included in this article.

## Author Contributions

YH contributed to conceptualization, funding acquisition methodology, supervision, writing, reviewing, and editing. JO contributed to data curation, investigation, project administration, resources, and writing original draft. HW contributed to database organization, software, visualization, and writing original draft. JZ contributed to methodology, software, statistical analysis, validation, writing, reviewing, and editing. AL contributed to equipment and software. XM contributed to investigation, writing reviewing, and editing. XD contributed to methodology, writing, reviewing, and editing. All authors contributed to the article and approved the submitted version.

## Conflict of Interest

The authors declare that the research was conducted in the absence of any commercial or financial relationships that could be construed as a potential conflict of interest.

## Publisher’s Note

All claims expressed in this article are solely those of the authors and do not necessarily represent those of their affiliated organizations, or those of the publisher, the editors and the reviewers. Any product that may be evaluated in this article, or claim that may be made by its manufacturer, is not guaranteed or endorsed by the publisher.
